# Short-Term Low-Carbohydrate High-Fat Diet in Healthy Young Males Renders the Endothelium Susceptible to Hyperglycemia-Induced Damage, An Exploratory Analysis

**DOI:** 10.3390/nu11030489

**Published:** 2019-02-26

**Authors:** Cody Durrer, Nia Lewis, Zhongxiao Wan, Philip N. Ainslie, Nathan T. Jenkins, Jonathan P. Little

**Affiliations:** 1 School of Health and Exercise Sciences, University of British Columbia Okanagan, Kelowna, BC V1V 1V7, Canada; cdurrer@live.com (C.D.); nialewis_26@hotmail.co.uk (N.L.); zhxwan@suda.edu.cn (Z.W.); philip.ainslie@ubc.ca (P.N.A.); 2 Department of Kinesiology, University of Georgia, Athens, GA 30602, USA; jenkinsn@uga.edu

**Keywords:** high-fat diet, microparticles, EMPs, flow-mediated dilation, FMD

## Abstract

Postprandial hyperglycemia has been linked to elevated risk of cardiovascular disease. Endothelial dysfunction and/or damage may be one of the mechanisms through which this occurs. In this exploratory study, we determined whether acute glucose ingestion would increase markers of endothelial damage/activation and impair endothelial function before and after a short-term low-carbohydrate high-fat diet (HFD) designed to induce relative glucose intolerance. Nine healthy young males (body mass index 23.2 ± 2 kg/m^2^) consumed a 75 g glucose drink before and <24 hours after consuming seven days of an iso-energetic HFD consisting of ~70% energy from fat, ~10% energy from carbohydrates, and ~20% energy from protein. CD31+/CD42b- and CD62E+ endothelial microparticles (EMPs) were enumerated at fasting, 1 hour (1 h), and 2 hours (2 h) post-consumption of the glucose drink. Flow-mediated dilation (FMD), arterial stiffness, and diameter, velocity, and flow of the common and internal carotid, and vertebral arteries were assessed in the fasting state and 1 h post glucose consumption. After the HFD, CD31+/CD42b- EMPs were elevated at 1 h compared to 2 h (*p* = 0.037), with a tendency for an increase above fasting (*p* = 0.06) only post-HFD. CD62E EMPs followed the same pattern with increased concentration at 1 h compared to 2 h (*p* = 0.005) post-HFD, with a tendency to be increased above fasting levels (*p* = 0.078). FMD was reduced at 1 h post glucose consumption both pre- (*p* = 0.01) and post-HFD (*p* = 0.005). There was also a reduction in FMD in the fasting state following the HFD (*p* = 0.02). In conclusion, one week of low-carbohydrate high-fat feeding that leads to a relative impairment in glucose homeostasis in healthy young adults may predispose the endothelium to hyperglycemia-induced damage.

## 1. Introduction

Impaired glucose tolerance and type 2 diabetes mellitus (T2DM) are associated with increased risk of cardiovascular disease (CVD; [1]) Accumulating evidence indicates that elevated postprandial hyperglycemia is an independent risk factor for CVD and cardiovascular mortality in individuals with, and without, T2DM [2]. The etiology of elevated postprandial hyperglycemia and increased cardiovascular risk has not been firmly established [3] but endothelial dysfunction is hypothesized to be the major mechanistic link [4]. Specifically, studies have shown that acute glucose infusion [5] or ingestion [6] can cause impairment in flow-mediated dilation (FMD) of the brachial artery in humans. Such impairment in endothelial function caused by acute glucose excursions appears to be exacerbated in conditions of glucose dysregulation, such that individuals with impaired glucose tolerance or T2DM experience a greater decline in FMD in response to glucose ingestion [7]. Experimental studies in humans [6] and mechanistic studies in cell culture [8] suggest that glycemic fluctuations may impair endothelial function by increasing oxidative stress and promoting an inflammatory response. As such, it is hypothesized that over time, repeated exposure to elevated postprandial hyperglycemia results in cumulative endothelial damage that contributes to increased risk of CVD. 

Although FMD is a well-established indicator of peripheral vascular function that is linked to CVD risk [9], it does not provide details into the cellular or molecular responses of endothelial cells. Endothelial microparticles (EMPs) are small (~100–1000 nm in diameter) vesicles shed from the plasma membrane of endothelial cells in response to activation, apoptosis, or damage. Circulating levels of EMPs are elevated in atherosclerosis, hypertension, T2DM, and metabolic syndrome [10], and as such are regarded as biomarkers for endothelial damage and dysfunction. EMPs can be characterized by the surface proteins associated with events triggering their release. CD31+/CD42b- EMPs are believed to be shed from apoptotic endothelial cells, whereas CD62E+ (E-selectin) EMPs indicate inflammatory activation of the endothelial cell of origin. Thus, measurement of circulating CD31+/CD42b- and CD62E+ EMPs can provide direct insight into damage and inflammation of the vascular endothelium [11].

The primary purpose of this exploratory investigation was to determine whether acute glucose ingestion would increase EMP release and impair FMD in humans. In order to perturb glucose tolerance, we studied the impact of a 75-gram oral glucose tolerance test (OGTT) drink before and after a 7-day low-carbohydrate high-fat diet (HFD) in young healthy male participants. Short-term HFDs have previously been shown to promote relative glucose intolerance in healthy human participants [12,13], and therefore allowed us to determine whether glucose ingestion impacted EMP release in the context of relative increase in postprandial hyperglycemia using a within-subjects design. This approach has the advantage of limiting the influence of baseline vascular dysfunction and endothelial damage that would have confounded a cross-sectional study comparing individuals of differing glucose tolerance status. Since high-fat feeding in animal models has been linked to endothelial damage and dysfunction [14], this design also provided the opportunity to determine the impact of short-term HFD on basal EMP levels and FMD. In addition, cerebral blood flow (CBF) is altered in response to HFDs in animals [15], however, data on extracranial CBF in humans is lacking. For this reason, we took this opportunity to assess CBF as an exploratory outcome.

## 2. Materials and Methods

### 2.1. Participants

Participants were enrolled in an interdisciplinary study that was also investigating the effects of high-fat feeding and glucose ingestion on inflammatory signalling and immune function in peripheral blood mononuclear cells [13,16]. Participants were excluded from the study if they (1) had a diagnosed metabolic disorder such as diabetes, metabolic syndrome, hypothyroidism, or any other condition known to affect metabolism; (2) had a history of mental health disorders such as depression, substance abuse, attention deficit hyperactivity disorder, or any other condition known to impact cognitive function; (3) had a history of inflammatory disorders such as rheumatoid arthritis, Crohn’s disease, irritable bowel syndrome, etc.; (4) had been prescribed any anti-inflammatory medication that they were unable to avoid for the duration of the study; (5) were already consuming a low-carbohydrate diet (e.g., “Atkins”, “Protein Power Plan”, “Paleo diet”, etc.); (6) had any dietary restrictions that would inhibit adherence to the study diet; (7) were unable to abstain from drugs (prescription and recreational) or alcohol for the duration of the study; (8) were unable to travel to and from the university in order to make their testing appointments; (9) had a body mass index (BMI: mass in kg divided by height in meters squared) > 30 kg/m^2^; (10) were not between the ages of 18–30 years old. All participants that were enrolled in the primary study also took part in this exploratory study. Recruitment was done via poster advertisement on the University campus and word of mouth. The study was fully explained to the participants prior to starting and written informed consent was obtained. Procedures were approved by the University of British Columbia Clinical Research Ethics Board. 

### 2.2. Study Design

The study involved three visits to the laboratory over approximately two weeks. On the first visit, the study was explained and written informed consent was obtained. Participants were provided with a 3-day food record detailing their typical diet during two weekdays and one weekend day (Table 1). Diet records were collected and analyzed prior to the second visit. During the second visit, all baseline measures were recorded. Participants reported to the lab for this visit following an overnight (≥8 hour) fast. Pre-testing included blood sampling and vascular function testing carried out in a fasted state and following consumption of a 75-gram oral glucose tolerance test (OGTT) drink. Following pre-testing, participants were provided with individualized meal plans and prepackaged food for the next seven days. Participants returned to the lab on the morning of the eighth day in a fasted state and repeated the pre-testing at the same time of day.

### 2.3. Diet

Total energy intake and macronutrient profile of participant’s usual diets were determined from the 3-day food records using FoodWorks Diet Analysis software (The Nutrition Company, Long Valley, NJ, USA). Individualized low-carbohydrate high-fat diets (HFD; supplying ~70% energy from fat, ~20% energy from protein, and ~10% energy from carbohydrates) isocaloric to usual intake (determined via 3-day diet records collected pre-HFD) were designed and prepared for each participant. Participants were provided with individualized meal plans and prepackaged food for the seven-day intervention and were instructed to only drink water during the study period. Participants were instructed to record their diet during the intervention and these records were analyzed post-HFD to confirm compliance (Table 1). Participants were instructed to maintain current levels of physical activity during the study.

### 2.4. Oral Glucose Tolerance Test

Following an overnight fast, participants reported to the laboratory for blood work and vascular function measurements. A baseline blood sample (pre-HFD) was obtained from an antecubital vein by venipuncture and collected into a 4-mL EDTA vacutainer tube (Becton-Dickinson, Franklin Lakes, NJ, USA). Baseline fasting vascular function tests were performed (see below) and participants were provided with a 75-gram oral glucose tolerance test (OGTT) drink (82.5 g dextrose monohydrate dissolved in 250 mL water). Blood samples were taken by venipuncture at 60 and 120 minutes post-OGTT. Tubes were placed on ice and, within 10 minutes, centrifuged for plasma collection at 1200 g for 15 minutes at 4 °C. 

### 2.5. Cardiovascular Measurements

#### 2.5.1. Blood Pressure and Heart Rate

Continuous beat-to-beat measures of arterial blood pressure (BP; finger photoplethysmography; Finapres Medical Systems, Biomedical Instruments, The Netherlands) and heart rate (HR; 3-lead ECG; ML132, ADInstruments, Colorado Springs, CO, USA) were recorded. Manual blood pressure recordings were taken at rest to calibrate the finger photoplethysmography measures. All data were sampled continuously using an analogue-digital converter (PowerLab/4S ML750; ADInstruments, Dunedin, New Zealand) interfaced with a computer and displayed in real time during testing. Data were stored for subsequent off-line analysis using commercially available software (Chart v7, ADInstruments, Dunedin, New Zealand). Baseline measures of BP and HR were averaged over 1 minute following 15-minutes of supine rest.

#### 2.5.2. Flow-Mediated Dilation

Brachial artery vascular function was assessed by endothelium-dependent FMD according to international guidelines [17]. A 10-MHz multifrequency linear array probe attached to a high-resolution ultrasound machine (Terason 3000^TM^, Teratech, Burlington, MA, USA) was used to image the brachial artery in the right arm. One minute of diameter and flow recordings preceded forearm cuff inflation (>200 mmHg) for 5 minutes. Diameter and flow recordings resumed 30 seconds prior to cuff deflation and continued for 3 minutes thereafter. 

Custom-designed edge-detection and wall-tracking software, which is largely independent of investigator bias, was utilised for the analysis brachial diameter and brachial blood flow velocity [17,18]. This software provides continuous and simultaneous diameter, velocity, and shear rate (SR; 4 x velocity/diameter) measurements, as well as post-hoc calculation of FMD and vasodilator capacity. This semi-automated software provides higher reproducibility of diameter measurements and reduces both observer error and bias with a reported intra-observer coefficient of variation (CV) for FMD% of 6.7% [18]. Data are presented as absolute (millimetres) and relative (percentage) rises from the preceding baseline diameter and are calculated based on standardized algorithms applied by the software [17]. In accordance with procedural recommendations [19,20,21], we also measured the post-deflation area under the shear rate curve in order to best interpret any changes in FMD. All FMD analyses were completed in a blinded fashion. 

#### 2.5.3. Extracranial Cerebral Blood Flow

Continuous diameter, velocity, and blood flow recordings in the right common carotid artery (CCA), right internal carotid artery (ICA), and right vertebral artery (VA) were obtained using a 10-MHz multifrequency linear array probe attached to a high-resolution ultrasound machine (Terason 3000, Teratech, Burlington, MA, USA). The CCA and ICA were measured at least 1.5–2 cm from the carotid bifurcation, whilst ensuring there was no evidence of turbulent or retrograde flow. The right VA was measured between the transverse process of C4 and the subclavian artery, but always at the same location within each subject. Average diameter and blood velocity recordings were made for ~30 seconds (see below), and care was taken to ensure probe position was stable so that the angle of insonation did not vary from 60 degrees. The sample volume was positioned in the centre of the vessel and adjusted to cover the width of the vessel diameter. Measurement settings for each extracranial artery within each individual were standardized for all measurement sets. 

All images were directly stored as an AVI file for offline analysis. Custom-designed edge-detection and wall-tracking software was utilised for the analysis of CCA, ICA, and VA diameter, velocity blood flow at 30 Hz [17]. Mean blood flow was determined as half the time averaged maximum velocity [18] multiplied by the cross-sectional lumen area. This method has been adopted previously by others and is used instead of the intensity weighted mean because the latter is more susceptible to noise and other distorting influences [22,23]. Global cerebral blood flow (CBF) was estimated assuming symmetrical bilateral flow in the ICA and VA [22,23] as: global CBF = (ICA_Flow_ + VA_Flow_) × 2. 

#### 2.5.4. Arterial Stiffness

Adhering to the international guidelines [24], hand held-tonometry (SPT-301 Millar Instruments, Houston, TX, USA) was employed to assess central (carotid-femoral pulse wave velocity; PWV) and peripheral (carotid-radial PWV) arterial stiffness. Twenty reproducible carotid-femoral artery waveforms and 20 separate carotid-radial artery waveforms were recorded simultaneously using mechanotransducers, which were applied directly to the skin and over the area of greatest pulsation. The distance from the sternal notch to the individual carotid, femoral, and radial artery pulse sites were measured along the surface of the body using a measuring tape. This technique was used as it has been shown to have the best agreement with aortic PWV measured invasively using cardiac catheterization [25]. The foot to foot method was used to determine pulse transit time, using a bandpass filter (5–30 Hz) to identify the foot or “notch” of the carotid-femoral and -radial waveform, and the difference in time from R interval to systolic upstroke at each location. Pulse distance was determined by subtracting the distance from carotid measurement to the sternal notch from the distance from the sternal notch to the femoral and radial pulse site measurement. Pulse-wave velocity was then determined by dividing distance by pulse transit time.

### 2.6. Plasma Analysis

#### 2.6.1. Glucose and Insulin

As previously reported [13], fingerstick blood glucose and plasma insulin was assessed throughout the OGTT. Fingerstick blood glucose was assessed following a ≥8-hour overnight fast immediately before and 15, 30, 60, and 120 minutes after consumption of the 75-g glucose drink using a OneTouch^®^ UltraMini^®^ meter (Lifescan, Milpitas, CA, USA). Insulin was assessed in plasma samples collected at fasting as well as 60 minutes and 120 minutes post-OGTT drink consumption via ELISA (Mercodia, Sweden) according to the manufacturer’s instructions. 

#### 2.6.2. EMPs

Circulating EMPs were measured in platelet poor plasma via flow cytometry (MACSQuant Analyzer, Miltenyi Biotec, Bergisch Gladbach, Germany) as described previously [26]. Frozen plasma samples were thawed at room temperature for 20 minutes and centrifuged at 1500 g for 15 minutes at room temperature. The top two-thirds of plasma were then further centrifuged again at 1500 g for 15 minutes to obtain platelet-poor plasma. The top 100 μL of platelet poor plasma was then incubated with fluorochrome labeled antibodies specific for CD62E (CD62E-phycoerythrin (PE), BD Biosciences (Mississauga, ON, Canada) Cat. No. 551145), CD31 (CD31-V450, BD Biosciences, Cat. No. 561653), and CD42b (CD42b-APC, BD Biosciences, Cat. No. 551061) for 20 minutes in the dark at 4 °C. Samples were then fixed with 93 μL of 2% paraformaldehyde and diluted up to 500 μL with sterile, 0.2-um filtered, phosphate buffered saline. A size gate was determined using 0.9 um National Institute of Standards and Technology-traceable polystyrene beads (Cat. No. 64019, Polysciences Inc., Warrington, PA, USA). Unstained and fluorescence minus one control were used to differentiate between true events and background/debris. EMPs were identified as CD62E+ and CD31+/CD42b- events within the microparticle size gate.

### 2.7. Data Analyses

Linear mixed effects analyses of the effect of HFD and glucose consumption on FMD, EMPs, glucose, insulin, and the vascular measures were performed using R [27] and the lme4 package [28]. As fixed effects, HFD and OGTT timepoint (glucose consumption) were entered into the model along with the interaction term. Random intercepts for subject were used. Visual inspections of residuals plots were used to assess homoscedasticity and normality. In instances where heteroscedasticity was noted, log-transformations of the data were used to satisfy this assumption. *p*-Values were obtained by likelihood ratio tests of the full models with the effect in question compared to models without the effect in question. All individuals were included in the analyses. Significant interactions and main effects of glucose consumption were followed up with Fisher least significant difference (LSD) post-hoc tests. Results are reported as means and standard deviations, or mean differences (fasting vs. post-OGTT or pre-HFD vs. post-HFD) with 95% confidence intervals. Cohen’s *d* effect sizes were calculated for significant pairwise comparisons. 

## 3. Results

### 3.1. Dietary Intervention

Nine healthy male subjects (21 ± 3 years, 76 ± 4 kg, 181 ± 9 cm, BMI 23.2 ± 2 kg/m^2^) participated in this study. All participants complied with the HFD intervention, as described previously [13].

### 3.2. Oral Glucose Tolerance Test

Fingerstick blood glucose and plasma insulin have been reported previously [13]. One week of HFD caused a relative impairment in glucose homeostasis in young healthy male subjects, as indicated by a significantly higher glucose area under the curve (AUC) in response to the 75-g OGTT drink following 7 days of HFD. Blood glucose measured at 30, 60, and 120 minutes post-OGTT were also significantly higher post-HFD when compared to baseline [13]. There was no effect of HFD on insulin during the OGTT. There was a main effect of time on plasma insulin with fasting (4.5 ± 1.6 mU/L), 60-minute (40.1 ± 26.7 mU/L), and 120-minute (22.8 ± 13.3 mU/L) post-OGTT consumption levels all being significantly different from each other (all *p* < 0.05).

### 3.3. Cardiovascular Measures

#### 3.3.1. Blood Pressure and Heart Rate

There were no effects of the diet or OGTT on systolic or diastolic blood pressure (Table 2). However, there were significant main effects of glucose consumption and HFD on mean arterial pressure. Mean arterial pressure was reduced (−3.2 mmHg, 95% CI [−6.3, −0.1], Cohen’s *d* = −0.44) at 60 minutes post-OGTT compared to fasting (*p* = 0.044). Mean arterial pressure was also reduced (−3.6 mmHg, 95% CI [−6.64, −0.47], Cohen’s *d* = −0.49) post-HFD compared to pre-HFD (*p* = 0.025). 

#### 3.3.2. FMD

**Figure 1:**
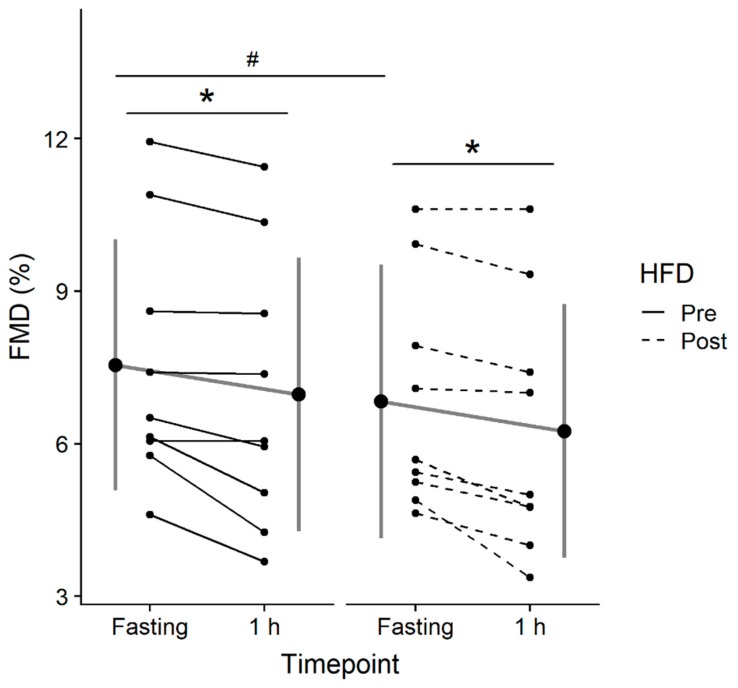
Flow-mediated dilation (FMD) assessed in the fasting state (Fasting) and 1-hour (1 h) following consumption of a 75-g glucose drink pre and post 7-day high fat diet (HFD). Data are expressed as the mean ± standard deviation (gray lines and large circles) and individually (solid and dashed black lines and small circles). Significant difference from Fasting to 1 h is denoted as * (*p* < 0.05), significant difference pre vs. post HFD is denoted as # (*p* < 0.05).

Baseline brachial artery diameters were similar prior to and after the HFD (pre-HFD: 0.41 cm, 95% CI [0.37, 0.44]; post-HFD: 0.42 cm, 95% CI [0.36, 0.47]; *p* = 0.62). In addition, there were no differences in shear rate area under the curve (SRAUC) post-HFD or post glucose consumption (both *p* > 0.05). The linear mixed-effects analysis revealed a significant effect of condition (*p* < 0.001) and time (*p* = 0.002) on FMD (Figure 1). Post-hoc tests were conducted to compare pre-HFD and post-HFD between timepoints, as well as pre-HFD vs. post-HFD at each timepoint. FMD showed a significant decrease pre-HFD (−0.58%, 95% CI [−0.18, −0.98], *p* = 0.01, Cohen’s *d* = −1.29), and post-HFD (−0.58%, 95% CI [−0.23, −0.93], *p* = 0.005, Cohen’s *d* = −1.12) in response to acute glucose ingestion. FMD was also lower post-HFD compared to pre-HFD in the fasting state (−0.71%, 95% CI [−0.16, −1.27], *p* = 0.02, Cohen’s *d* = −0.75) and had a tendency to be lower post-OGTT (−0.72%, 95% CI [0.015, −1.45], *p* = 0.053, Cohen’s *d* = −0.99).

#### 3.3.3. Extracranial Cerebral Blood Flow

There were no differences in blood flow in CCA, ICA, or VA (all *p* > 0.05). Accordingly, global CBF was also unaltered by the diet intervention or acute OGTT (*p* > 0.05). There was a significant effect of time for ICA diameter (*p* = 0.01), VA diameter (*p* = 0.005), and CCA diameter (*p* = 0.044), which revealed that ICA, VA, and CCA diameters were all larger (ICA: 0.017 cm, 95% CI [0.0044, 0.029], Cohen’s *d* = 0.52; VA: 0.015 cm, 95% CI [0.0049, 0.026], Cohen’s *d* = 0.60; CCA: 0.012 cm, 95% CI [0.00031, 0.024], Cohen’s *d* = 0.18) at 60 minutes vs. fasting. There was no effect of HFD on any vessel diameters (all *p* > 0.05). There were no differences in either CCA or VA velocity, however, there was a main effect of time for ICA velocity, where velocity was slower at 60 minutes compared to fasting (−3.33 cm/s, 95% CI [−5.98, −0.69]; *p* = 0.015, Cohen’s *d* = −0.08).

#### 3.3.4. Arterial Stiffness

There was a significant main effect of diet for peripheral PWV (*p* = 0.027), with lower PWV post-HFD (−0.47 m∙s^−1^, 95% CI [−0.89, −0.06], Cohen’s *d* = −0.48). Central PWV was unaltered by diet (*p* = 0.15) and both central and peripheral PWV were unaltered by the OGTT (*p* > 0.05).

### 3.4. EMPs

**Figure 2:**
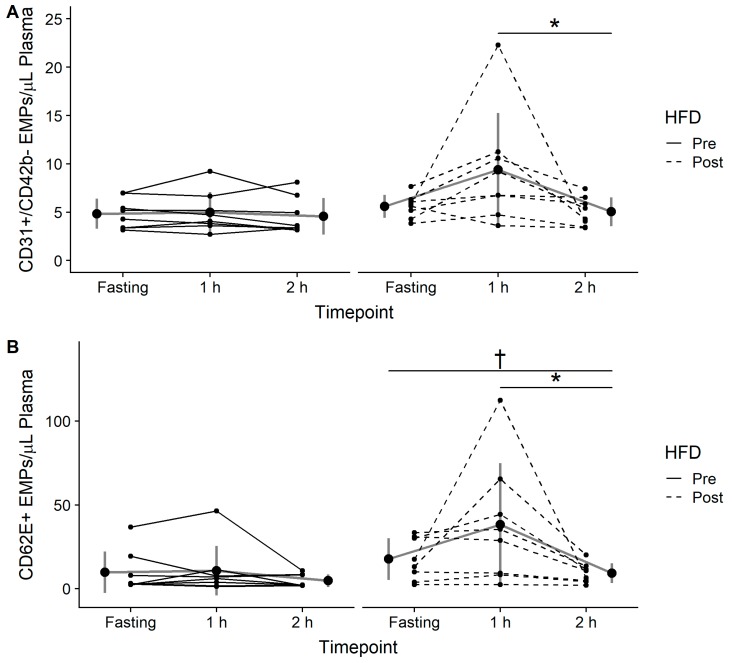
(**A**) CD31+/CD42b- endothelial microparticles (EMPs) and (**B**) CD62E+ EMPs assessed in the fasting state (Fasting), 1-hour (1 h), and 2-hours (2 h) following consumption of a 75-g glucose drink pre and post 7-day high-fat diet (HFD). Data are expressed as the mean ± standard deviation (gray lines and large circles) and individually (solid and dashed black lines and small circles). Significant difference from 1 h to 2 h post-HFD is denoted as * (*p* < 0.05), significant difference from fasting to 2 h post-HFD is denoted as † (*p* < 0.05).

There was a significant effect of time and diet for both CD31+/CD42b- (time: *p* = 0.03; diet: *p* = 0.003) and CD62E (time: *p* = 0.02; diet: *p* < 0.001) EMPs. Post-hoc analysis revealed that there was a tendency for CD31+/CD42b- EMPs to be higher at 60 minutes when compared to fasting (95% CI [−1.10, 6.71], *p* = 0.06, Cohen’s *d* = 0.68) and that CD31+/CD42b- EMPs were significantly higher at 60 minutes when compared to 120 mins post-OGTT (95% CI [1.10, 9.70], *p* = 0.037, Cohen’s *d* = 0.72) in the post-HFD condition only (Figure 2A). CD62E EMPs followed the same pattern, with a tendency towards higher levels at 60 minutes when compared to fasting (95% CI [−1.21, 16.5], *p* = 0.078, Cohen’s *d* = 0.59) and significantly higher levels at 60 minutes compared to 120 minutes (95% CI [2.77, 57.85], *p* = 0.005, Cohen’s *d* = 0.84; Figure 2B). CD62E EMPs were also significantly lower at 120 minutes compared to fasting in both the pre-HFD (95% CI [−1.21, −5.89], *p* = 0.02, Cohen’s *d* = −0.54) and post-HFD (95% CI [−1.13, 10.37], *p* = 0.03, Cohen’s *d* = −0.8) conditions. There were no correlations between the change in FMD and the changes in either CD31+/CD42b- or CD62E EMPs (data not shown, all *p* > 0.05).

## 4. Discussion

The main findings of the present study are that the one-week low-carbohydrate high-fat diet, which causes relative glucose intolerance (as we reported previously; [13]), coincides with a reduction in FMD in the fasting state and following ingestion of glucose. Furthermore, the consumption of a HFD for one week led to increased levels of endothelial damage markers (CD31+/CD42b- and CD62E+ EMPs) during a physiological excursion into hyperglycemia. These findings indicate that a short-term HFD in young healthy men (i) can reduce FMD; and (ii) may render the endothelium susceptible to hyperglycemia-induced damage. We also report findings on the impact of glucose ingestion on extracranial CBF, with the main findings indicating that an acute excursion into hyperglycemia induced increases in ICA, VA, and CCA diameter with a corresponding reduction in velocity in ICA but no statistically significant changes in flow in any of these vessels. 

Our results indicate that FMD is attenuated in healthy young men after both an excursion into hyperglycemia and following consumption of the HFD for one week. However, despite the induction of relative glucose intolerance with the HFD, there were no synergistic (i.e., interactive) effects between the two, as the consumption of 75 g of glucose led to a similar reduction in FMD pre- and post-HFD. It is well established that FMD is reduced after consumption of an OGTT drink [7,29] and a single high-fat meal in humans [30]. Furthermore, short-term high-fat diets are often used in animal studies to induce endothelial dysfunction [31]. However, research examining FMD following short-term low-carbohydrate high-fat diet interventions in young healthy human populations is lacking. Longer duration low-carbohydrate high-fat diet interventions have demonstrated increases [32], reductions [33], and no change [34] in FMD, but these studies contain confounding factors, most importantly co-existing weight loss and caloric restriction. This inconsistency in the literature makes it difficult to interpret the effect of repeated high-fat feeding on FMD in the absence of weight loss. Our findings demonstrate that a short-term HFD leads to a reduction in FMD, similar to the effect on FMD following a single high-fat meal. It has previously been suggested that the impairment in FMD following the consumption of a high-fat meal could be attributed to heightened oxidative stress and reduced nitric oxide (NO) bioavailability [35]. 

It is well established that acute hyperglycemia impairs endothelial function [7,29], which is also suggested to be mediated by increased oxidative stress and reduced NO bioavailability [36]. A recent meta-analysis investigating the acute effects of meal consumption on FMD reported an average reduction of ~2% in postprandial FMD [37]. Our findings, although demonstrating a smaller decrease in postprandial FMD (0.58%), are similar to the literature in that respect. Following the HFD, glucose levels were higher after the OGTT, but the hyperglycemia-induced reduction in FMD was not exacerbated (i.e., no interaction effects). This is perhaps suggestive of separate mechanisms leading to the fasting reduction in FMD following the HFD, and the acute hyperglycemia-induced depression in FMD. While the clinical relevance of these small reductions in FMD cannot be determined by the present study in young healthy males, it is possible that the increase in risk would be even greater for an already at-risk population. It is important to note that baseline brachial artery diameter and SRAUC were unchanged throughout the study, indicating that the changes observed in FMD were not due to these variables.

Microparticles (MP) are defined as submicron vesicles that are shed from the plasma membranes of various cell types in response to activation, injury, and/or apoptosis [10]. Although initially regarded as cellular debris [38], MPs are now recognized to play important physiological roles such as signalling molecules (reviewed in [39]). MPs, including EMPs, can contain various biologically active molecules such as proteins, cytokines, mRNAs, or microRNAs [40]. Expressed at the surface of MPs are most of the membrane-associated proteins of their parent cells, making flow cytometry a viable method for the detection and differentiation of these particles. It has been suggested that EMPs are markers of damage, with CD62E+ EMPs being indicative of inflammatory activation and CD31+/CD42b- EMPs indicative of apoptosis [10]. There seems to be a link between hyperglycemia and circulating EMPs, given the elevated levels reported in T2DM patients [41]. Animal models have demonstrated that EMPs generated under high-glucose conditions can induce vascular inflammation and impair endothelial function, whereas EMPs generated from healthy endothelial cells do not [42]. For this reason, we hypothesized that EMPs would increase following an excursion into hyperglycemia in humans and that this might be related to endothelial function as measured by FMD. While we did observe a reduction in FMD and an increase in circulating EMPs following consumption of a glucose load both pre- and post-HFD, there was no correlation between EMPs and FMD. In addition, high-fat meals have also been shown to increase circulating EMPs [43]; however, we did not observe an increase in basal EMPs following the HFD, despite the reduction in FMD. This would indicate that the EMPs were not directly responsible for the impairment in FMD, but rather both are mediated by other, possibly separate, mechanisms. It also appears that the impact of the HFD, in terms of endothelial damage markers, was only revealed when combined with hyperglycemia. This suggests that consuming a HFD over the short-term predisposes the endothelium to hyperglycemia-induced damage. 

We observed a significant increase in diameter in the ICA, VA, and CCA following consumption of a 75-gram glucose drink, with no significant increase in flow in any of these vessels. It has previously been reported that ICA diameter has been shown to increase with elevations in circulating insulin concentration [44]. Indeed, there was a 10-fold increase in insulin concentration during the OGTT when the increases in diameter were measured. A reduction in velocity was detected in the ICA, which explains the consistent ICA flow. However, we did not detect statistically significant reductions of velocity in the VA or CCA or in their respective blood flow. This is likely due to the relatively small sample size and resultant insufficient power to detect such changes. Nonetheless, there appeared to be no direct effects of the short-term HFD on basal CBF in young healthy men. The implications of more chronic changes in glucose and insulin on cerebrovascular health and remodelling require future study. 

We also observed a modest reduction in mean arterial pressure 60 minutes post glucose consumption and a reduction in the fasting state following the HFD. It is possible that the reduction following glucose consumption is related to splanchnic blood pooling, which is commonly seen in the postprandial state [45]. The lower mean-arterial pressure seen post-HFD could be due to a reduction in sympathetic tone. The finding of reduced peripheral PWV after the HFD supports this view, however, without a direct measure of sympathetic tone in this, this remains speculative.

## 5. Conclusions

In conclusion, one week of high-fat, low-carbohydrate feeding that leads to a relative impairment in glucose homeostasis in healthy young adults may predispose them to hyperglycemia-mediated endothelial damage as well as a reduction in endothelial function. The findings also suggest that a short-term HFD and acute glucose excursions may reduce FMD via separate and non-synergistic mechanisms. Increased susceptibility of the endothelium to hyperglycemia-induced damage provides evidence that the combination of a HFD with glucose ingestion could be detrimental to vascular health. These findings are especially relevant given the recent increase in popularity of low-carbohydrate, high-fat diets. These new findings suggest that if young, healthy males are following such diets, a temporary lapse in adherence with consumption of a food causing a glucose spike might lead to acute endothelial damage.

## Figures and Tables

**Table 1:** Pre- and post-HFD (high-fat diet) macronutrient and energy intake.

Variable	Pre-HFD	Post-HFD
Energy intake (kcal/day)	2584 ± 456	2417 ± 267
Fat intake (% daily energy)	37 ± 6	71 ± 0.6
Carbohydrate intake (% daily intake)	46 ± 9	11 ± 0.9
Protein intake (% daily intake)	17 ± 3	18 ± 0.6
kcal, kilocalories		

**Table 2:** Cardiovascular measures before and after a one-week HFD both fasting and 60 minutes (1 h) post-OGTT.

	Pre-HFD		Post-HFD		*p*-Value		
Variable	Fasting	1 h	Fasting	1 h	Time	Condition	Interaction
Systolic Blood Pressure (mmHg)	120.2 ± 10	116.7 ± 11.6	117.3 ± 9	113.8 ± 7.9	0.07	0.14	1
Diastolic Blood Pressure (mmHg)	72.2 ± 7.6	67.8 ± 8.5	66.9 ± 8.5	65.3 ± 7.2	0.14	0.06	0.46
Mean Arterial Pressure (mmHg)	88.2 ± 5.4	84.1 ± 6.5	83.7 ± 7.8	81.5 ± 6.6	0.04	0.03	0.52
Resting Heart Rate (BPM)	60.0 ± 9.5	59.7 ± 12.2	56.7 ± 7.7	60.8 ± 8.9	0.11	0.35	0.05
Central PWV (m/s)	5.7 ± 0.5	5.8 ± 0.4	5.6 ± 0.8	5.4 ± 0.5	0.59	0.16	0.34
Peripheral PWV (m/s)	7.8 ± 1.5	7.4 ± 1.2	7.1 ± 0.6	7.2 ± 1.1	0.53	0.03	0.26
CCA Diameter (cm)	0.68 ± 0.03	0.70 ± 0.03	0.69 ± 0.04	0.70 ± 0.04	0.04	0.96	0.71
CCA Velocity (cm/s)	35.52 ± 6.82	35.06 ± 4.20	34.27 ± 3.86	33.48 ± 4.97	0.61	0.22	0.94
CCA Flow (mL/s)	6.70 ± 1.22	6.90 ± 0.80	6.54 ± 1.07	6.50 ± 1.06	0.53	0.23	0.68
ICA Diameter (cm)	0.53 ± 0.03	0.55 ± 0.04	0.52 ± 0.04	0.54 ± 0.03	0.01	0.06	0.53
ICA Velocity (cm/s)	38.61 ± 7.62	35.19 ± 3.80	38.11 ± 7.79	34.86 ± 4.87	0.02	0.75	0.95
ICA Flow (mL/s)	4.35 ± 1.09	4.15 ± 0.82	4.00 ± 0.87	3.97 ± 0.62	0.41	0.06	0.53
VA Diameter (cm)	0.40 ± 0.04	0.42 ± 0.04	0.39 ± 0.04	0.41 ± 0.04	0.005	0.10	0.38
VA Velocity (cm/s)	19.86 ± 4.53	19.40 ± 3.94	20.33 ± 3.26	20.02 ± 3.50	0.41	0.24	0.87
VA Flow (mL/s)	1.32 ± 0.51	1.37 ± 0.53	1.26 ± 0.39	1.36 ± 0.40	0.16	0.46	0.60

mmHG, millimeters of mercury; PWV, pule wave velocity; BPM, beats per minute; m/s, meters per second; cm, centimeters; cm/s, centimeters per second; mL/s, milliliters per second; CCA, common carotid artery; ICA, internal carotid artery; VA, vertebral artery.

## References

[1] Kannel W.B., McGee D.L. (1979). Diabetes and glucose tolerance as risk factors for cardiovascular disease: The Framingham study. Diabetes Care.

[2] O’Keefe J.H., Bell D.S. (2007). Postprandial hyperglycemia/hyperlipidemia (postprandial dysmetabolism) is a cardiovascular risk factor. Am. J. Cardiol..

[3] Borch-Johnsen K., Neil A., Balkau B., Larsen S., Nissinen A., Pekkanen J., Tuomilehto J., Jousilahti P., Lindstrom J., Pyorala M. (2001). Glucose tolerance and cardiovascular mortality-Comparison of fasting and 2-hour diagnostic criteria. Arch. Intern. Med..

[4] Williams S.B., Goldfine A.B., Timimi F.K., Ting H.H., Roddy M.-A., Simonson D.C., Creager M.A. (1998). Acute hyperglycemia attenuates endothelium-dependent vasodilation in humans in vivo. Circulation.

[5] Ceriello A., Esposito K., Piconi L., Ihnat M.A., Thorpe J.E., Testa R., Boemi M., Giugliano D. (2008). Oscillating glucose is more deleterious to endothelial function and oxidative stress than mean glucose in normal and type 2 diabetic patients. Diabetes.

[6] Ceriello A., Taboga C., Tonutti L., Quagliaro L., Piconi L., Bais B., Da Ros R., Motz E. (2002). Evidence for an independent and cumulative effect of postprandial hypertriglyceridemia and hyperglycemia on endothelial dysfunction and oxidative stress generation: Effects of short-and long-term simvastatin treatment. Circulation.

[7] Kawano H., Motoyama T., Hirashima O., Hirai N., Miyao Y., Sakamoto T., Kugiyama K., Ogawa H., Yasue H. (1999). Hyperglycemia rapidly suppresses flow-mediated endothelium-dependent vasodilation of brachial artery. J. Am. Coll. Cardiol..

[8] Karbach S., Jansen T., Horke S., Heeren T., Scholz A., Coldewey M., Karpi A., Hausding M., Kröller-Schön S., Oelze M. (2012). Hyperglycemia and oxidative stress in cultured endothelial cells—A comparison of primary endothelial cells with an immortalized endothelial cell line. J. Diabetes Complicat..

[9] Green D.J., Jones H., Thijssen D., Cable N., Atkinson G. (2011). Flow-mediated dilation and cardiovascular event prediction: does nitric oxide matter?. Hypertension.

[10] Chironi G.N., Boulanger C.M., Simon A., Dignat-George F., Freyssinet J.-M., Tedgui A. (2009). Endothelial microparticles in diseases. Cell Tissue Res..

[11] Jenkins N.T., Padilla J., Boyle L.J., Credeur D.P., Laughlin M.H., Fadel P.J. (2013). Disturbed blood flow acutely induces activation and apoptosis of the human vascular endothelium. Hypertension.

[12] Lovejoy J.C., Windhauser M.M., Rood J.C., de la Bretonne J.A. (1998). Effect of a controlled high-fat versus low-fat diet on insulin sensitivity and leptin levels in African-American and Caucasian women. Metabolism.

[13] Wan Z., Durrer C., Mah D., Simtchouk S., Robinson E., Little J.P. (2014). Reduction of AMPK activity and altered MAPKs signalling in peripheral blood mononuclear cells in response to acute glucose ingestion following a short-term high fat diet in young healthy men. Metabolism.

[14] Ketonen J., Pilvi T., Mervaala E. (2010). Caloric restriction reverses high-fat diet-induced endothelial dysfunction and vascular superoxide production in C57Bl/6 mice. Heart Vessel..

[15] Freeman L.R., Haley-Zitlin V., Rosenberger D.S., Granholm A.-C. (2014). Damaging effects of a high-fat diet to the brain and cognition: a review of proposed mechanisms. Nutr. Neurosci..

[16] Wan Z., Durrer C., Mah D., Simtchouk S., Little J.P. (2014). One-week high-fat diet leads to reduced toll-like receptor 2 expression and function in young healthy men. Nutr. Res..

[17] Black M.A., Cable N.T., Thijssen D.H., Green D.J. (2008). Importance of measuring the time course of flow-mediated dilatation in humans. Hypertension.

[18] Woodman R., Playford D., Watts G., Cheetham C., Reed C., Taylor R., Puddey I., Beilin L., Burke V., Mori T. (2001). Improved analysis of brachial artery ultrasound using a novel edge-detection software system. J. Appl. Physiol..

[19] Pyke K.E., Tschakovsky M.E. (2007). Peak vs. total reactive hyperemia: which determines the magnitude of flow-mediated dilation?. J. Appl. Physiol..

[20] Pyke K.E., Tschakovsky M.E. (2005). The relationship between shear stress and flow-mediated dilatation: Implications for the assessment of endothelial function. J. Physiol..

[21] Atkinson G., Batterham A.M., Black M.A., Cable N.T., Hopkins N.D., Dawson E.A., Thijssen D.H., Jones H., Tinken T.M., Green D.J. (2009). Is the ratio of flow-mediated dilation and shear rate a statistically sound approach to normalization in cross-sectional studies on endothelial function?. J. Appl. Physiol..

[22] Willie C., Macleod D., Shaw A., Smith K., Tzeng Y., Eves N., Ikeda K., Graham J., Lewis N., Day T. (2012). Regional brain blood flow in man during acute changes in arterial blood gases. J. Physiol..

[23] Willie C.K., Smith K.J., Day T.A., Ray L.A., Lewis N.C., Bakker A., Macleod D.B., Ainslie P.N. (2013). Regional cerebral blood flow in humans at high altitude: gradual ascent and 2 week at 5050 m. J. Appl. Physiol..

[24] Laurent S., Cockcroft J., Van Bortel L., Boutouyrie P., Giannattasio C., Hayoz D., Pannier B., Vlachopoulos C., Wilkinson I., Struijker-Boudier H. (2006). Expert consensus document on arterial stiffness: methodological issues and clinical applications. Eur. Heart J..

[25] Weber T., Ammer M., Rammer M., Adji A., O’rourke M.F., Wassertheurer S., Rosenkranz S., Eber B. (2009). Noninvasive determination of carotid–femoral pulse wave velocity depends critically on assessment of travel distance: a comparison with invasive measurement. J. Hypertens..

[26] Durrer C., Robinson E., Wan Z., Martinez N., Hummel M.L., Jenkins N.T., Kilpatrick M.W., Little J.P. (2015). Differential impact of acute high-intensity exercise on circulating endothelial microparticles and insulin resistance between overweight/obese males and females. PLoS ONE.

[27] R Core Team (2018). R: A Language and Environment for Statistical Computing.

[28] Bates D., Maechler M., Bolker B., Walker S. (2015). Fitting Linear Mixed-Effects Models Using lme4. J. Stat. Softw..

[29] Mah E., Noh S.K., Ballard K.D., Matos M.E., Volek J.S., Bruno R.S. (2011). Postprandial Hyperglycemia Impairs Vascular Endothelial Function in Healthy Men by Inducing Lipid Peroxidation and Increasing Asymmetric Dimethylarginine: Arginine–3. J. Nutr..

[30] Cuevas A.M., Guasch V., Castillo O., Irribarra V., Mizon C., San Martin A., Strobel P., Perez D., Germain A.M., Leighton F. (2000). A high-fat diet induces and red wine counteracts endothelial dysfunction in human volunteers. Lipids.

[31] Kobayasi R., Akamine E.H., Davel A.P., Rodrigues M.A., Carvalho C.R., Rossoni L.V. (2010). Oxidative stress and inflammatory mediators contribute to endothelial dysfunction in high-fat diet-induced obesity in mice. J. Hypertens..

[32] Volek J.S., Ballard K.D., Silvestre R., Judelson D.A., Quann E.E., Forsythe C.E., Fernandez M.L., Kraemer W.J. (2009). Effects of dietary carbohydrate restriction versus low-fat diet on flow-mediated dilation. Metabolism.

[33] Phillips S.A., Jurva J.W., Syed A.Q., Syed A.Q., Kulinski J.P., Pleuss J., Hoffmann R.G., Gutterman D.D. (2008). Benefit of low-fat over low-carbohydrate diet on endothelial health in obesity. Hypertension.

[34] Keogh J.B., Brinkworth G.D., Clifton P.M. (2007). Effects of weight loss on a low-carbohydrate diet on flow-mediated dilatation, adhesion molecules and adiponectin. Br. J. Nutr..

[35] Bae J.-H., Bassenge E., Kim K.-B., Kim Y.-N., Kim K.-S., Lee H.-J., Moon K.-C., Lee M.-S., Park K.-Y., Schwemmer M. (2001). Postprandial hypertriglyceridemia impairs endothelial function by enhanced oxidant stress. Atherosclerosis.

[36] Ceriello A., Esposito K., Piconi L., Ihnat M., Thorpe J., Testa R., Bonfigli A.R., Giugliano D. (2008). Glucose “peak” and glucose “spike”: Impact on endothelial function and oxidative stress. Diabetes Res. Clin. Pract..

[37] Thom N., Early A., Hunt B., Harris R., Herring M. (2016). Eating and arterial endothelial function: A meta-analysis of the acute effects of meal consumption on flow-mediated dilation. Obes. Rev..

[38] Hargett L.A., Bauer N.N. (2013). On the origin of microparticles: From “platelet dust” to mediators of intercellular communication. Pulm. Circ..

[39] Hoyer F.F., Nickenig G., Werner N. (2010). Microparticles–messengers of biological information. J. Cell. Mol. Med..

[40] Mause S.F., Weber C. (2010). Microparticles: protagonists of a novel communication network for intercellular information exchange. Circ. Res..

[41] Rautou P.-E., Vion A.-C., Amabile N., Chironi G., Simon A., Tedgui A., Boulanger C.M. (2011). Microparticles, vascular function, and atherothrombosis. Circ. Res..

[42] Jansen F., Yang X., Franklin B.S., Hoelscher M., Schmitz T., Bedorf J., Nickenig G., Werner N. (2013). High glucose condition increases NADPH oxidase activity in endothelial microparticles that promote vascular inflammation. Cardiovasc. Res..

[43] Harrison M., Murphy R.P., O’Connor P.L., O’Gorman D.J., McCaffrey N., Cummins P.M., Moyna N.M. (2009). The endothelial microparticle response to a high fat meal is not attenuated by prior exercise. Eur. J. Appl. Physiol..

[44] Chaudhuri A., Kanjwal Y., Mohanty P., Rao S., Sung B.H., Wilson M.F., Dandona P. (1999). Insulin-induced vasodilatation of internal carotid artery. Metabolism.

[45] Jansen R.W., Penterman B.J., Van Lier H.J., Hoefnagels W.H. (1987). Blood pressure reduction after oral glucose loading and its relation to age, blood pressure and insulin. Am. J. Cardiol..

